# Local Field Potentials: Myths and Misunderstandings

**DOI:** 10.3389/fncir.2016.00101

**Published:** 2016-12-15

**Authors:** Oscar Herreras

**Affiliations:** Department of Translational Neuroscience, Cajal Institute-CSICMadrid, Spain

**Keywords:** local field potentials, EEG, volume-conduction, spatial discrimination, spontaneous activity, network oscillations, neuronal circuits, cell assembly

## Abstract

The intracerebral local field potential (LFP) is a measure of brain activity that reflects the highly dynamic flow of information across neural networks. This is a composite signal that receives contributions from multiple neural sources, yet interpreting its nature and significance may be hindered by several confounding factors and technical limitations. By and large, the main factor defining the amplitude of LFPs is the geometry of the current sources, over and above the degree of synchronization or the properties of the media. As such, similar levels of activity may result in potentials that differ in several orders of magnitude in different populations. The geometry of these sources has been experimentally inaccessible until intracerebral high density recordings enabled the co-activating sources to be revealed. Without this information, it has proven difficult to interpret a century's worth of recordings that used temporal cues alone, such as event or spike related potentials and frequency bands. Meanwhile, a collection of biophysically ill-founded concepts have been considered legitimate, which can now be corrected in the light of recent advances. The relationship of LFPs to their sources is often counterintuitive. For instance, most LFP activity is not local but remote, it may be larger further from rather than close to the source, the polarity does not define its excitatory or inhibitory nature, and the amplitude may increase when source's activity is reduced. As technological developments foster the use of LFPs, the time is now ripe to raise awareness of the need to take into account spatial aspects of these signals and of the errors derived from neglecting to do so.

## The many faces of field potentials

The fluctuation of field potentials (FPs) in the brain is an aspect of neural activity that is increasingly used as a reflection of the ongoing transmission through neural networks. In addition, FPs seed the electroencephalograms (EEG) that are recorded from outside the brain and thus, they have enormous clinical relevance. Identifying the cellular origin of these potentials is therefore an issue of fundamental importance in Neuroscience. Although the biophysics underlying these events has been well established, there does not seem to be a consensus as to how this information should be used and interpreted. Indeed, FPs are an epiphenomenon of electrical activity in cell aggregates and as such, spatiotemporal fluctuations may either have a strong relationship or no relationship to the activity in the contributing unit sources. A number of structural and functional micro—and mesoscopic factors combine differently in each structure to shape FPs and to influence the information contained therein. In cases, it is the morphological constraint of the neuron sources or the population architecture that define these features. Also, FPs may reflect the dynamics of single cells, or this may be completely occluded and it is the assembly firing that they reflect. In addition, FPs may replicate the activity of nearby cells or of remote populations. It is therefore essential to consider all the relevant factors together. Otherwise, the lessons drawn from FPs in one structure are likely to lead to errors when applying the same set of rules to interpret FPs in others.

### Technological developments and the need for biophysical training

In recent years, technological advances have made the recording equipment necessary to study FPs much more amenable to researchers from different backgrounds. Some of the most recent developments will ultimately make intracranial monitoring of FPs a non-invasive approach (Seo et al., 2015), and a further boost for the use of FPs may just be around the corner. Unfortunately, equipment does not come with manuals as to how to interpret brain signals. Perhaps for this reason, many issues related to FPs have for decades waned between the technical accounts of expert biophysicists and the pragmatic interpretations of other researchers. In addition to the already complex spatial treatment of large and distant sources needed to account for EEG recordings at the scalp (Nunez and Srinivasan, 2006), microscopic factors should also now be considered since they are essential to understand the so-called intracerebral local field potentials (LFPs). Unfortunately, adequate experimental and technical approaches to deal with the spatial nature of intracranial FPs have been lacking until recently, meaning that researchers have only been able to focus on their temporal fluctuations. Through repetition, a number of guidelines for the interpretation of LFPs have become established as undisputed rules amongst non-experts. Nonetheless, the fact that these fail to take into account spatial factors has led to widespread and important misconceptions. Consequently, while FPs in different structures may indeed reflect different aspects of neural activity, in many cases the differences proposed simply reflect incorrect interpretations. Indeed, a number of apparent contradictions with other modalities of functional or anatomical data have emerged that seriously compromise the reputation of LFPs as a reliable quantitative estimation of neuron activity.

The aim of this paper is to raise awareness of this issue by listing the most common misinterpretations that arise from neglecting micro—and mesoscopic spatial factors in LFPs. For each of these, I will provide an informal explanation as well as the types of scientific problems and questions to which they apply. Needless to say that a number of the issues relating to FPs are yet to be resolved. However, for a more profound or formal analysis of the issues raised here, monographic and more specialized texts can be consulted (Lorente de Nó, 1947; Woodbury, 1960; Rall and Shepherd, 1968; Elul, 1971; Lopes da Silva and Van Rotterdam, 1982; Gloor, 1985; López-Aguado et al., 2001; Nunez and Srinivasan, 2006; Nelson et al., 2008; Lindén et al., 2011; Herreras et al., 2015).

It is also worth noting that the problems inherent to surface (EEG) recordings, though closely related to intracerebral ones, must be treated somewhat differently due to the particular set of technical limitations associated with them. Nevertheless, a more thorough understanding of how spatial microscopic factors affect intracerebral LFPs will also help clarify certain aspects of the EEG as a mass phenomenon, and this should be considered a necessary step toward accurately interpreting these recordings. Such information is also likely to be beneficial to the ongoing debate into other specific issues, such as the different information gathered from FPs observed over different spatial scales (Nunez et al., 1997; Foster et al., 2016). Indeed, not all types of neurons, pathways or structures are appropriate to generate LFPs and hence, they will not contribute to EEGs either.

### Cellular basis, geometry, volume conduction, cancellation

**Figure 1:**
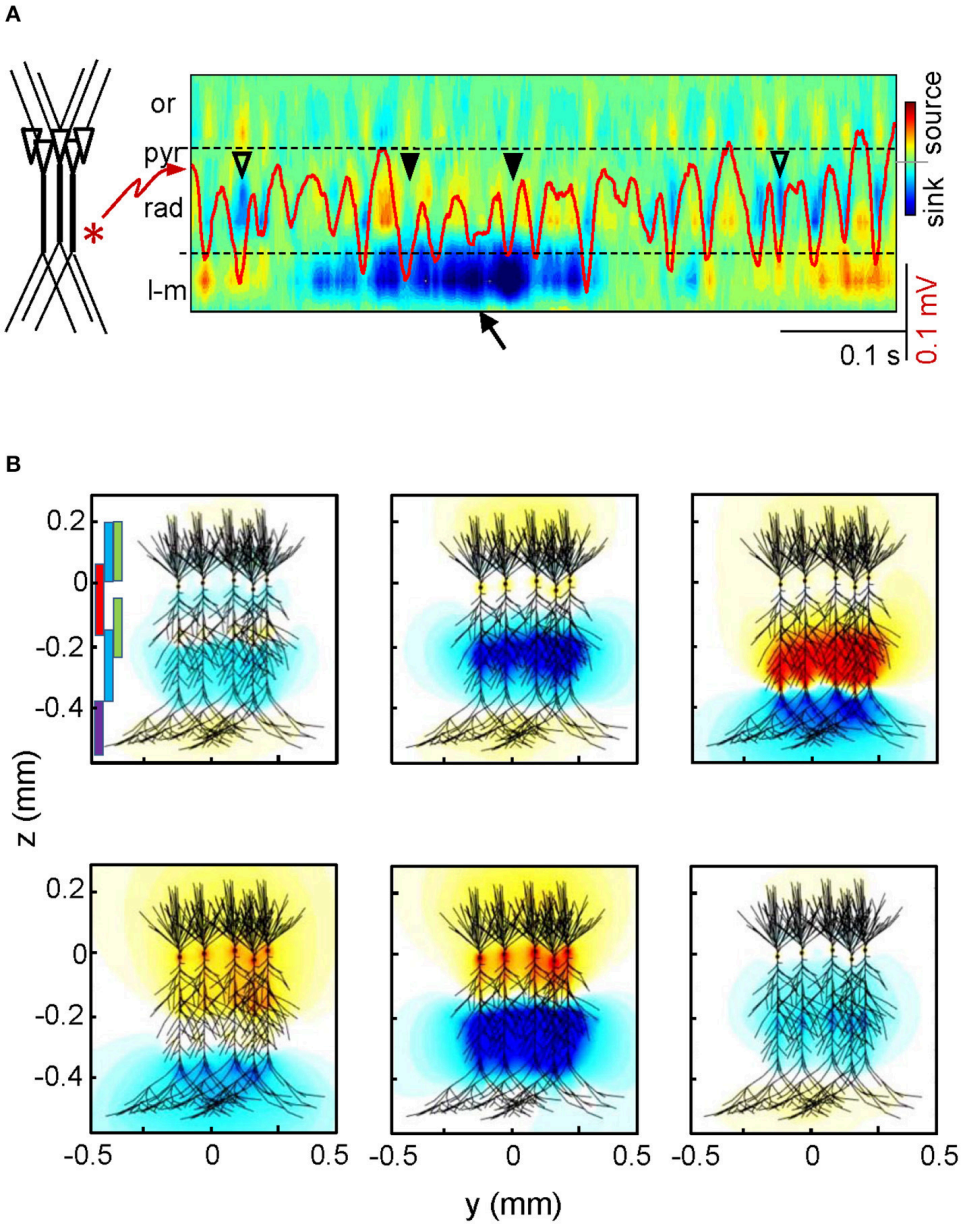
**The same neurons produce countless different sources of current. (A)** Spatiotemporal map of current sources and sinks obtained for a sample LFP epoch in the hippocampal CA1 region (the superimposed red trace is recorded in the st. radiatum). The large magnitude of the currents elicited by an input at distal dendrites (arrow) distorts the magnitude of currents produced by an adjacent oscillatory input at gamma frequency. Note the poor matching of the magnitude of currents and LFP gamma waves. Or, pyr, rad, l-m: strata oriens, pyramidale, radiatum, and lacunosum-moleculare. **(B)** The panels represent two-dimensional snapshots of computed field potentials generated in a volume by different combinations of synaptic inputs onto a realistic aggregate of hippocampal pyramidal cells, mimicking the spontaneous activity in the intact animal. The synaptic territories of afferent pathways are depicted by colored bars in the upper left panel (purple: distal excitation from entorhinal cortex; red: perisomatic basket cell inhibition; green: commissural excitatory input, blue: Schaffer excitatory input). Negative and positive potentials are coded in blue and yellow-red, respectively. Reproduced with permission from Martín-Vázquez et al. (2013) and Herreras et al. (2015). See animated reproduction in Video 1.

When discussing the cellular basis of certain FP events or oscillations, we normally refer to the type of neuron or the population that generates the underlying electrical currents. However, identifying the physical substrate of a source is not trivial, and the reconstruction of its precise geometry becomes unmanageable. A short historical note may help us focus on this issue. The theoretical basis underlying FPs is well-known and its quantitative application to intracerebral FP recordings began in the 1940's when Lorente de Nó (1947) proposed Maxwell's quasistatic approach to the distribution of current flow in a volume conductor. It was soon patent that the main problem was the highly irregular geometry and the microscopic nature of the current sources (the neurons), which could be combined into countless spatial configurations on account of their variable co-activation. Just one simple detail illustrates the complexity of the problem: although a single neuron has a stable geometry it may operate as many different sources of current *with varying geometry* depending on the subgroup of co-activated synapses at each instant (Figure 1 and Video 1).

Determining the elementary units of LFPs is therefore somewhat tricky (see Elul, 1971). Establishing the physical boundaries of the source of current is obviously a point of departure, although the distribution of charge within those physical limits is even more relevant. Conveniently, much neural processing is carried out by neuron aggregates or assemblies whose units are often co-activated, either by natural stimuli or because they constitute a functional processing aggregate that is molded by use (experience). Only then, and provided that, such assemblies fulfill strict anatomo-functional criteria, can their transmembrane currents build measurable FPs in the extracellular space. Since neuron assemblies are activated by other assemblies through a common axon bundle that enters a distinct synaptic territory of the target neurons, the FPs generated have a pathway-specificity that can be taken advantage of given their unique spatial distribution (Herreras et al., 2015). Nevertheless, it follows that each neuron/population originates as many geometrically different sources of current as afferent pathways. Although these are not excessive in number, their independent activation will cause the synaptic currents to mix in a variable manner and produce countless different voltage shells in a short period of time (see Figure 1 and Video 1). On the experimental side, this makes gathering data over repeated trials impractical and the search for adequate solutions requires collecting (instantaneous) spatial profiles of the voltage. Moreover, the interpretation of such three-dimensional voltage shells is complicated by certain factors. One is that currents may not only sum but they may also cancel out and as such, estimating them from voltage profiles contributed by multiple sources (e.g., the current source density-CSD) gives no hint as to the actual magnitude and composition since the extent of cancellation is unknown (see Figure 1A). Also, currents produce field potentials that do not remain local but rather, they extend unevenly throughout the volume far from the sources. Consequently, any combination of synaptic currents raises a FP that has different amplitude and possibly a distinct polarity at different sites. If the contributions to such mixtures vary with the location, which site should we choose to establish quantitative relationships with any other physiological or diagnostic parameter? Thus, it is imperative to disentangle the contribution of all the sources to the voltage recorded at any site, be they distant or local.

Understanding the role of volume conduction on the recording of so-called far fields (see The “Reach” of LFPs Does Not Depend on the Amplitude at Its Origin) goes hand in hand with the comprehension of how the structural features of the sources define the spatial cancellation of currents within their own boundaries. This is perhaps the most critical and neglected concept that we shall attempt to clarify below. Indeed, in the best of all cases, only a minimal fraction of the total current survives cancellation within cell-sized volumes. There is much active debate into the nature of LFPs, and issues, such as the frequency-dependent filtering properties of the tissue, the contribution of spikes, the participation of non-neuronal sources and that of intrinsic currents have often been discussed in contemporary monographs (Bédard et al., 2004; Buzsáki et al., 2012; Reimann et al., 2013; Halnes et al., 2016). However, in these forums the structural factors that ultimately determine how a source is recorded locally or from a distance are barely mentioned. No doubt, all these issues fade in relevance when we consider that most pathways do not produce any significant FP, whatever the strength or the frequency of the underlying synaptic currents, or their role in neural processing. To exemplify this, I will briefly summarize our present understanding of the low-pass filtering properties of the nervous tissue, introducing its dependence on source geometry as a new element for debate (see Tissue Capacitance, Monopoles, Frequency-Filtering, and Source Geometry).

### The power of semantics: local field potentials are barely local

While the term *local* is admittedly confusing, a consensus has been reached amongst experimenters to use the term *local field potential* for signals recorded by small intracerebral electrodes, as opposed to those obtained with the large electrodes commonly used in surface EEG recordings (Buzsáki et al., 2012). Such an apparently innocuous semantic assignment may be the origin of a common erroneous association between the electrode tip size (and other associated properties like the impedance) and “sensitivity” to temporal features of the signal, whereby small-tipped electrodes are thought to pick up more local activity and higher frequencies than large electrodes. Neither of these assumptions is strictly correct even though they may often appear supported by facts (see Electrodes Have No Spatial Sensitivity or Frequency Selectivity). Besides, it is important to keep in mind that the proportion of local and remote contributions at any site cannot be ascertained from a single recording point. Indeed, in many brain structures most of the FP activity actually comes from remote sites since the local generators are weak (Martín-Vázquez et al., 2016). This is critical in studies of functional connectivity for which single-site recordings are strongly discouraged.

A common mistake is to consider that since the electric field drops drastically from a point source, that associated to brain sources must also extend over short distances and hence, FPs should be very local. As reasoned below, this does not count for the complex brain sources whose FP rate of decay is jointly governed by the spatial extension and the internal distribution of charges within the source. These concepts relate to an issue that raises considerable debate, as discussed below (see The “Reach” of LFPs Does Not Depend on the Amplitude at Its Origin), which is the extent and functional meaning of cortical receptive fields for sensory inputs measured through LFPs or spikes.

Comparing ground-referenced recordings with recordings differentiated between two nearby sites provides an excellent quantitative measurement of how local the contribution to LFPs really is. Recordings between two electrodes separated by 100 μm yield FP amplitudes 2–3 orders of magnitude smaller than each of the single-ended ones (i.e., recorded against a ground electrode or a far neutral reference). Accordingly, either the bulk of the FP is contributed by sources located further away than that distance (maximum far contribution) or the source is larger than the electrode separation and both electrodes are contained within it (Robinson, 1980). *Sensu stricto*, LFPs are anything but local, and it is necessary to more thoroughly explore the FPs in search of the spatial gradients around the electrodes to clarify whether the activity comes from homologous neurons in the same structure or from remote sites. The fact that LFPs are recorded intracerebrally does not guarantee that the sources are any closer to electrodes.

## The evil triad: baseline, polarity and a cocktail party

There are three main problems that hamper the interpretation of intracerebral FPs in terms of the activity contributed by the active units. Moreover, it is necessary to develop an integrated understanding of each of these issues as they interact tightly, and thus, neglecting one of them will distort the interpretation of the other two.

**Figure 2:**
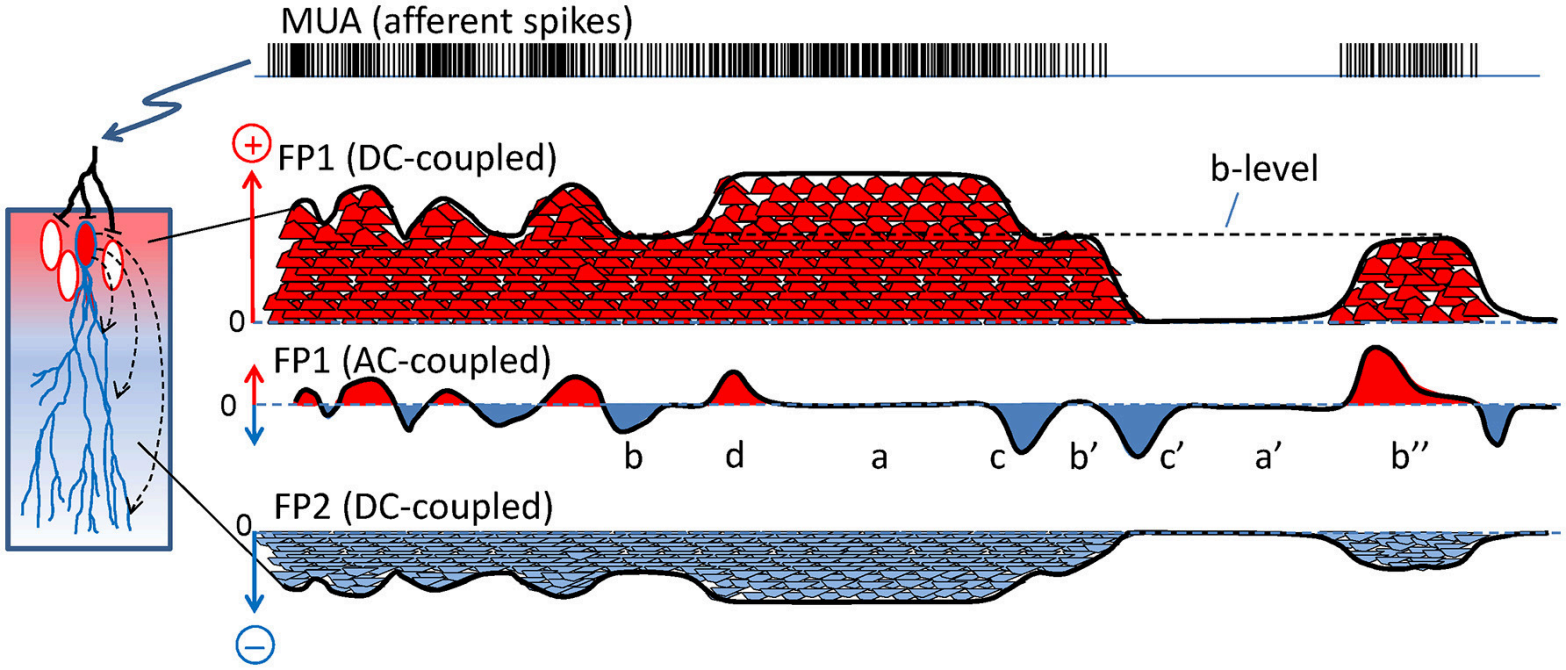
**The scaling of dipolar currents to field potentials is distorted by AC coupling**. Scheme representing how a train of spikes (multi-unit activity, MUA) afferent to a neuron population (left) produces a compound FP. Each spike produces an elementary synaptic current (small red and blue bumps) that sum in the extracellular space to others. The currents leave (red) or enter the cells (blue). For simplification, the FP (black line) is recreated only at two locations (FP1 and FP2) when recorded in DC-coupled mode, and only one is represented in AC-coupled mode (FP1). In the DC-mode, the FP1 and FP2 have mirror time courses and site-specific unique polarity. In the AC-coupled mode the slow (DC) components are removed and the FP is zeroed (the new baseline is artificial). The remaining fluctuations still preserve a similar time-course as the “skyline” of the native DC-potentials but suffer different and important distortions compared to the underlying currents: a and a′ denote epochs of similar flat FPs for large or null currents; b, b′, and b″ denote peaks in the FP of different polarity for similar levels of current (dashed line marked b-level); and c and c′ mark two FP waves with similar features that actually correspond to very different transients of the current. Also, net positive charge can be recorded as positive or negative FP values in different instants and sustained changes become reflected by short waves (d and c′).

Problem #1: the baseline. A major technical concern is the lack of a true baseline in the recordings obtained with standard AC-coupled amplifiers (Martín-Vázquez et al., 2013). This approach removes the DC-components and provokes the artificial zeroing of the signal, converting FP waves of a given polarity into a collection of smaller positive and negative half-waves (Brankačk et al., 1993) whose relation to the afferent activity is therefore severely distorted, leading to spurious correlations with other physiological markers. For those who have never considered the functional relevance of the DC component when inferring the properties and nature of a FP, this concept and some of the errors derived from neglecting it are illustrated in Figure 2. For instance, in epochs of intense sustained activity (i.e., current density) the FPs may be rendered as small as in epochs of minimal activity (compare a/a′ in Figure 2). Also, similar levels of activity may be correlated with transients of either positive or negative FP polarity, or even those of null value (b/b′/b″). FP waves of similar amplitude may encode totally different changes of activity (c/c′). Finally, sustained changes of activity go unnoticed as only the transients are reflected in AC-coupled recordings (d). Unfortunately, the DC component cannot be easily recovered (Hartings et al., 2009), and only when the FP fluctuations are known to be contributed by a single source (Problem #3) is there a chance of recovering the “skyline” potential and the correct polarity of the fluctuations (Problem #2: Martín-Vázquez et al., 2013).

**Figure 3:**
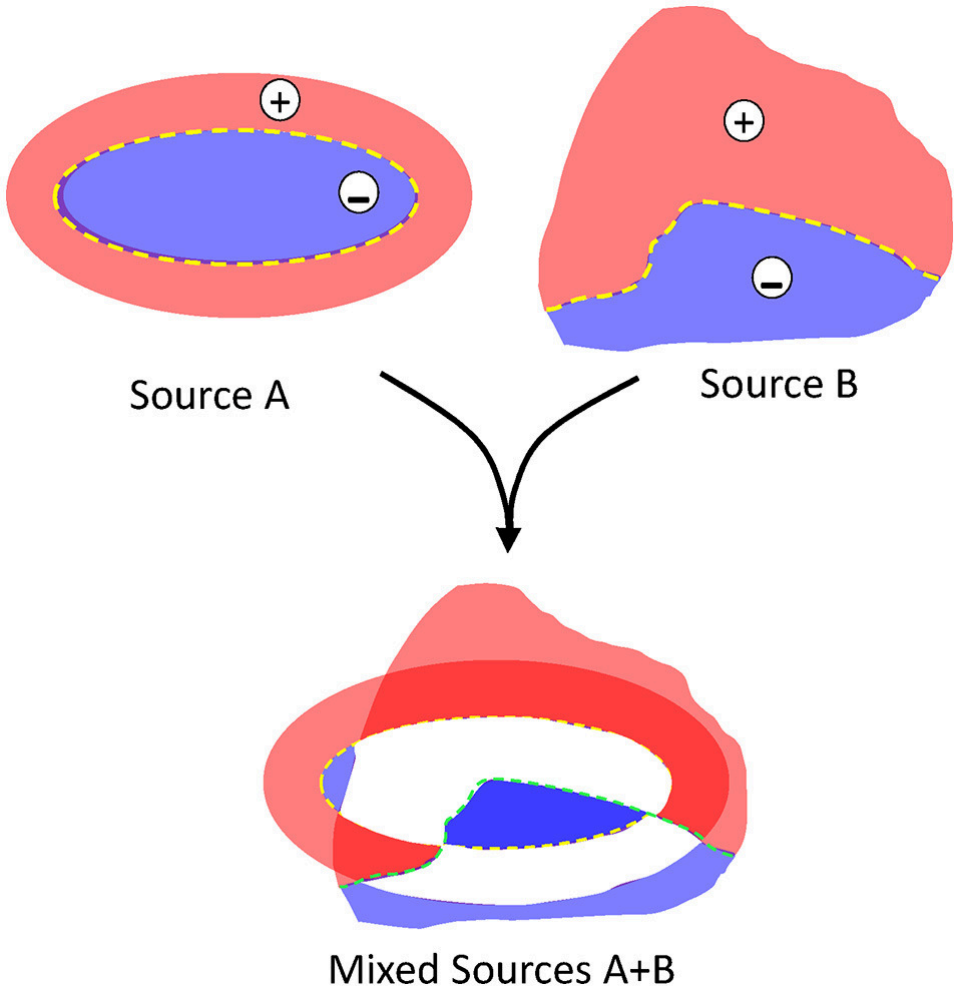
**The blending of multiple dipolar sources produces spatial indetermination of the contributions**. Schematic representation of two different sources blending in a given space. For isolated sources, the positive and negative areas are well delimited by the structural factors and all values are proportional but for blended sources (as in the LFP), the quantitative contribution of the originals cannot be estimated: white areas may adopt any positive or negative value at different instants, and darkened red and blue areas adopt values in different sites that are not proportional between them.

Problem #2: polarity. Due to charge conservation, neuronal current sources have a dipolar nature, i.e., as much current enters as leaves a cell at any instant. Consequently, inward and outward currents must be spatially displaced for FPs to build up. The bulk of the microscopic synaptic currents are canceled out by the spatial overlap of inward and outward currents from the same or nearby cells. Yet, in the few instances when the subcellular, cellular and macroscopic geometry jointly promote the segregation of zones where currents are not balanced, the inward and outward surges (sinks and sources) typically have a different spatial distribution. As such, the positive and negative FP zones have an asymmetric non-intuitive spatial distribution (Woodbury, 1960; see Figure 3). Due to technical problem #1 (Figure 2), the polarity recorded is unreliable as it may be artefactually reversed by the zeroing effect of AC-coupled recordings in some instances but not in others. In addition, given the multiple source origin (see Problem #3 below), it is impossible to assign positive or negative fluctuations to any of the mixing contributions (Figure 3).

Problem #3: multiple origins. Spontaneous FPs are typically generated by a cocktail of synaptic currents elicited by diverse afferent pathways at different sites or distinct target populations. At successive time points, the relative contribution of the different sources to any point in space varies, making it impossible to determine either the time course or the gross characteristics of any of the contributing sources. As a case in point, the vast literature on oscillatory LFPs (such as the theta or gamma rhythms in the cortex and hippocampus) is very heterogeneous, probably reflecting the different recording strategies, data analyses and biophysical background of those performing the analysis. In addition, the more abundant irregular LFPs are poorly studied even though they reflect the stochastic nature of incoming natural stimuli (Bullock et al., 2003). Only in a few cases, when the contribution of a source is much larger than that of all the others, are quantitative estimations obtained from raw LFPs reasonably assigned to a pathway. Even then, identifying the origin and target populations may be an arduous task. Thus, in raw multisource LFPs recorded in AC-coupled mode it is impossible to estimate when one or another contribution varies and to what extent (Problem #1). One might expect as many different contributions to LFPs as anatomical pathways with distinct synaptic territories, which for a simple region like the hippocampal CA1 may be around 20 (Somogyi and Klausberger, 2005; Takács et al., 2012). Conveniently, there are several geometrical limiting factors and very rarely do they all favor the spatial segregation of positive and negative charges, which means that the magnitude of the respective dipoles ranges over several orders of magnitude. Thus, only a few pathways render measurable potentials in the order of tenths to hundredths of microvolts (Korovaichuk et al., 2010; Benito et al., 2014). Even then, one can easily imagine that it may not be possible to attribute positive or negative FP transients to local excitatory or inhibitory synaptic currents, or to passive ones from an adjacent synaptic territory (Problems #1, #2). It is therefore absolutely necessary to disentangle the temporal fluctuations produced by each of the contributing pathways in order to establish a quantitative relationship with the activity of a single afferent population. So far, the only approaches providing acceptable separation that maintains complete temporal resolution of the mixing sources are the spatial discrimination techniques (Makarova et al., 2011; Fernández-Ruiz and Herreras, 2013; Martín-Vázquez et al., 2013, 2016; Benito et al., 2014, 2016; Głąbska et al., 2014; Schomburg et al., 2014).

## Common misconceptions around local field potentials

It is important now to clarify some of the common misconceptions brought about by neglecting spatial factors as the essential determinants of FP amplitude and extension, although the following is not an exhaustive list explaining all such situations.

### The dynamics of LFPs reflects the activity of afferent populations and not that of the source neurons

A common misconception is to consider that the fluctuations in LFPs reflect the temporal pattern of spike activity of the neurons near the electrodes. Actually, FPs are also present in regions in which the units are not firing, such as when incoming synaptic inputs remain subthreshold, or when they are dominated by inhibitory currents. Alternatively, there may be no such inputs at all and the FPs recorded are volume-conducted from far off sources. It should be reminded that the current underlying FPs is produced by the so-called source neurons, while the dynamics of such current is established by the incoming series of spikes emitted by *other neurons*, which may (e.g., interneurons) or may not (e.g., projection cells from distant nuclei) be located nearby (Herreras et al., 2015). Consequently, one shouldn't expect much of a temporal relationship between LFPs and spikes recorded by the same electrode. Indeed, the spikes emitted by the source neurons are the result of the integration of many synaptic inputs, some of which contribute to LFPs but others may not, and it may be some of these later inputs those driving cell firing (see Rasch et al., 2008).

In the literature, unit-to-LFP relationships are often established or dismissed without strict reference to causality, or one is assumed on the erroneous ground regarding the origin of the LFPs. This may explain cases in which a firing unit relates to a given LFP in some tasks but not others (Donoghue et al., 1998; Canolty et al., 2012). It is necessary to locate the afferent neurons setting the synaptic inputs in the zone where the LFP is recorded, and not doing so may lead to erroneous inferences on the functional connectivity between structures. For instance, it is common to read that the hippocampus is functionally connected to one structure or another, or associated to a behavioral task, based on observations that relate theta rhythm with other parameters at other sites. However, it would be more accurate to propose that a relationship exists between one structure and that which sets up the hippocampal theta, i.e., the medial septum. Thus, medial septal GABAergic neurons projecting to the hippocampus fire rhythmically at a theta frequency (Borhegyi et al., 2004; Simon et al., 2006) and they set-up theta LFPs there, either directly by activating synaptic currents or resonant properties in postsynaptic cells, or indirectly through interposed local interneurons (Colgin, 2013; Tsanov, 2015). In turn, most hippocampal projection cells, which generate the theta currents, become silent themselves during theta-related behavior, while only a few (place cells) fire transiently at a theta frequency. In simple words, there is a strong possibility that the LFP activity recorded in a given structure reflects the activity of other structures (cf. in Figure 1 of Herreras et al., 2015).

### LFP amplitude is poorly related to the magnitude of the synaptic inputs

**Figure 4:**
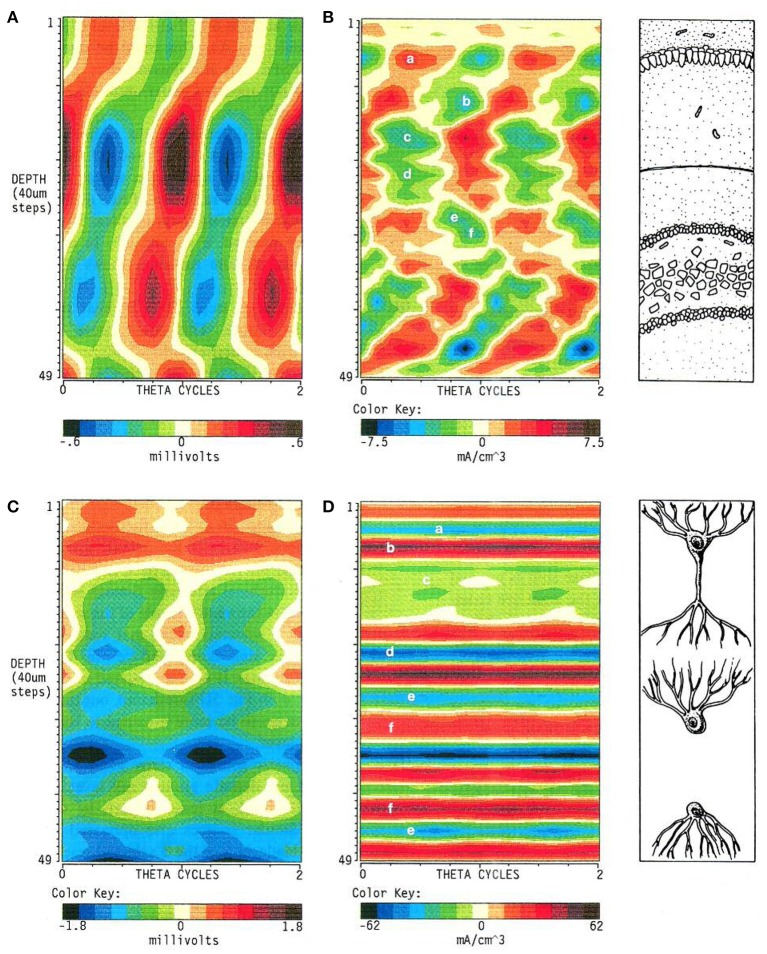
**Recording in AC- or DC-coupled mode produces entirely different spatiotemporal maps of LFPs**. The figure illustrates the striking differences in the LFPs **(A,C)** and the corresponding currents **(B,D)** estimated from their voltage gradients when the presence of sustained components contributing to LFPs are considered **(C,D)**. These are rejected by AC-coupled amplifiers and the resultant potentials become zeroed, creating spurious alternations of polarity (oscillations) from essentially sustained potentials. Reproduced with permission from Brankačk et al. (1993).

The changes in amplitude of LFPs over time are commonly taken as matching variations of the activity they are supposed to reflect (i.e., the currents). It is indeed intuitive that an increase in the synchronization of synaptic currents should render larger extracellular potentials, as we learnt from stimulus-evoked responses. However, a number of technical issues and experimental situations make this assumption untenable for LFPs (see The Evil Triad: Baseline, Polarity, and a Cocktail Party). Some counterintuitive discrepancies arise between the amplitude of potentials and currents, mostly because of the lack of a true (DC) baseline (Figure 2). While AC-coupling barely modifies evoked potentials, it has a large impact on LFPs. A typical example is the completely different spatiotemporal map of hippocampal theta rhythm obtained through AC- or DC-coupled recordings (Figure 4, Brankačk et al., 1993). In standard AC-coupled recordings, some sites display positive-negative alternation of voltage, while the CSD analysis of the spatial gradients yields alternating sequences of current sources and sinks. However, alternation is largely occluded when considering the DC component and it gives way to a dominant negative potential. In turn, the currents estimated take the form of a sustained plateau that is only inward or outward in a given strata. Besides, it is some eight times larger than AC-estimated currents. Consequently, the interpretation of the cellular elements responsible for the production of the current and the afferent pathways that provide the synaptic inputs differ.

Another major factor hindering an estimation of the magnitude of the current is the varying composition of the contributing sources over time. Since currents are added linearly in the extracellular space, the FP produced by each source serves as a variable offset to all others (Figure 3) and hence, the resulting FP may grow or reduce through changes in a source at the same place or nearby (depending on the site, extension, polarity, etc.). Without a precise description of the FP gradients in the space, this problem cannot be resolved. FP transients are far more reliable in pathway-specific components that are disentangled from LFPs (Makarov et al., 2010; Herreras et al., 2015), since in these, the spatial dimension is set aside and they are free from the varying contributions of nearby (or remote) sources. Using this approach, we and others found a mutual distortion amongst the multiple sources of gamma oscillations in the Dentate Gyrus/CA1 border that arise from independent synaptic inputs to granule and pyramidal cells (Benito et al., 2014, 2016; Schomburg et al., 2014). Such gamma sources co-activate often but with variable phase coupling. When they are generated by two inputs to the same neurons, both the currents and the LFPs become distorted (Benito et al., 2016). However, when the gamma inputs enter different nearby populations, only LFPs get distorted (in amplitude and phase) through volume-conduction. Also, slower distal sources in the CA1 pyramidal cells offset the net magnitude of the currents associated to other CA1 gamma sources (Martín-Vázquez et al., 2013). In addition, concurrent (phase-coupled) gamma sources in the CA1 and CA3 pyramidal cells modify each other's features in function of the orientation of the respective dipoles. For example, Schaffer gamma LFPs in the CA1 increase in magnitude, and change their spatial profile and site of reversal (Martín-Vázquez et al., 2016). So far, such interactions have been studied only in the hippocampus, but they can be expected for any other structure.

### LFP polarity is not related to the chemical nature of synaptic currents

It is commonly assumed that negative potentials reflect depolarization, while positive potentials reflect hyperpolarizing currents. However, the FP sources of neuronal origin all have a dipolar nature, and they create distinct zones of positive and negative polarity (Figure 3; Woodbury, 1960). These are rarely symmetrical in the space, and that which shall become dominant and extend further depends primarily on spatial factors, namely the subcellular distribution of synaptic currents, and the 3D arrangement and the collective architecture of the co-activated cells (see below). Furthermore, the dominant polarity does not reflect the active synaptic currents. For instance, in simple aggregates like the orderly population of hippocampal granule cells, both somatic inhibition and dendritic excitation produce positive FP transients with similar spatial distribution (Fernández-Ruiz et al., 2013), and they are undistinguishable in wide areas. In one case, the dominant positivity is created by the active inhibitory synaptic currents, while in the other it is generated by the passive share of the excitatory current loops (both outward in nature). Additional techniques should be employed to safely determine the nature of the LFP, using pharmacological tools (e.g., neurotransmitter blockers), genetically encoded probes for ion monitorization, or optogenetic tools (Gorostiza et al., 2013). Moreover, to avoid multisynaptic confounders it is safer to study their effects on isolated pathway-specific components of the LFP (e.g., Martín-Vázquez et al., 2013).

### LFP frequency bands do not characterize pathways, populations or synaptic events

It is common practice to filter LFP signals in order to highlight the “activities of interest”. From pioneering EEG studies that reported the association between certain brain oscillations and behavioral states or tasks (e.g., Dement and Kleitman, 1957; Vanderwolf, 1969), a still widespread notion has survived that considers frequency bands as physiological entities. Indeed, oversimplification may link them to specific structures, populations, neurons or synaptic events, although the relevant evidence is incomplete and therefore, circumstantial. The concept of LFP oscillations is commonly oversimplified, mostly due to the neglect of the problems mentioned above (The Evil Triad: Baseline, Polarity, and a Cocktail Party), and to the excessive emphasis on the temporal analogies between the behavior of a single unit and LFPs without paying due consideration to the proper spatial dimension of one and the others. In general, temporal relations between unitary spikes or intracellular events to LFPs cannot be used to establish the cellular basis of the later (Elul, 1971). The different cellular elements involved, and essential differences of the factors that govern one and the other (units vs. population) make their observed relations occasional and subjected to interpretation. The variety of such shortcomings is large, and here I shall only refer to a few (see Fernández-Ruiz and Herreras, 2013, and Martín-Vázquez et al., 2013 for additional discussion).

It should first be considered that the vast majority of central neurons that show oscillatory discharge patterns also fire in other modes (Ranck, 1973; Vinogradova, 2001), and the transitions between them obey the demands of processing that are largely unpredictable. It is therefore intuitive that the same cells contribute to compound oscillatory LFPs as much as to irregular ones or to “invisible” DC-like potentials. Thus, band selection eliminates an important fraction of the information contained in LFPs, if not the majority (Figure 2; Bullock et al., 2003).

Importantly, oscillatory LFPs may arise from the cyclic firing of neurons in synchrony or simply, from adequate windowing of irregularly firing neurons (e.g., firing windows established by rhythmic inhibition). While in the first case, the oscillation is observed both in the unit and the compound LFP, in the latter it is not, and therefore a lack of correlation may be erroneously assumed. The opposite may also be true, i.e., that a rhythmic discharge of units is erroneously considered the origin of a concomitant LFP oscillation that is actually generated by other local network or by remote cells in another structure. A paradigmatic case that has been rapidly assimilated is the widespread belief that gamma oscillations are inhibitory (Buzsáki and Wang, 2012). It is important to discriminate the mechanism that sets the oscillation from the current that produces the LFP. Gamma disruption using GABA-receptor blockers or genetic interference, or the visualization of gamma firing in interneurons, or the presence of inhibitory oscillations in the membrane potential of units in phase with the field oscillations, provide circumstantial evidence of the influence of inhibition on such events. Nevertheless, these data do not resolve the chemical nature of the synaptic current or the identity of the target cell that originates the extracellular currents (see The Dynamics of LFPs Reflects the Activity of Afferent Populations and Not That of the Source Neurons). Indeed, none of these observations rule out cases where interneurons set the clock for excitatory neurons to build LFP oscillations in their targets, such as was found for those mediated by the Schaffer or the lateral and medial Perforant Pathways in the CA1 and Dentate Gyrus (Fernández-Ruiz et al., 2012; Benito et al., 2014; Schomburg et al., 2014). In these cases, the origin of oscillatory LFPs is in excitatory neurons of the CA3 and entorhinal cortex, respectively, or through the excitatory recurrent pathway in the CA3 itself (Martín-Vázquez et al., 2016). These excitatory gamma oscillations are phase locked with others of truly GABAergic origin in the same region with which they blend in native LFPs (ibid.; Benito et al., 2016). However, the inhibitory pacemaker units do not necessarily provoke an LFP in the principal cells, either because the extracellular currents undergo extensive cancellation or because they are too small compared to other local sources with a more favorable geometry. For instance, several subtypes of interneurons with distinct phase coupling to gamma rhythms provide synaptic input to CA1 pyramidal cells (Hájos et al., 2004; Varga et al., 2014), while only one consistent inhibitory gamma generator is found in this subfield (Benito et al., 2014). Also, when both excitatory and inhibitory oscillations occur, and the respective sources are located close to each other, it is not easy to separate them and this requires the use of spatial discrimination techniques to isolate the respective electric fields (Herreras et al., 2015; Benito et al., 2016). It is essential to understand that there are many anatomical inputs whose synaptic currents may or may not be seen intracellularly in soma impalements, while their contribution to external population currents (and LFPs) may be large and is determined by the subcellular distribution of the activated synaptic territory, as well as other mesoscopic factors.

Regarding the exploration of the cellular basis of oscillatory LFPs, it is increasingly common practice to simplify the preparation in order to apply techniques or paradigms that are not readily implemented in the intact animal. In such cases, the role of natural networks in establishing rhythms is substituted by external manipulations that may instigate the oscillatory behavior of intrinsic channels or local circuits that are normally occluded or driven by exogenous inputs with a similar or totally different timing. Thus, despite successfully achieving LFP oscillations at the same frequency, the pharmacology and/or the spatial distribution often differs from that in the *in vivo* situation. For instance, the theta rhythm in the hippocampus is known to peak and originate from synaptic currents in the stratum lacunosum-moleculare *in vivo* (Brankačk et al., 1993; Benito et al., 2014), while the distinct spatial distribution in other preparations (e.g., Goutagny et al., 2009) suggests that the current sources differ. With regards to LFP generators, similar is not identical and thus, the spatial distribution must be carefully assessed.

A classic problem of LFP filtering is the statistical bias produced in studies seeking the participation of units on network (LFP) oscillations. Very often, a large fraction of the spikes are unrelated to the dominant LFP frequency and they contribute to the so-called background firing. As a result the significance in relation to the oscillatory LFP may be destroyed (e.g., in histograms, auto- and cross-correlations, LFP-spike averages, etc.). Conversely, a significant relationship may overstress the oscillatory influence due to the lack of other frequency components in filtered LFPs. For instance, in the mentioned case of theta oscillations in the hippocampus (Figure 4) one might expect that the cells of origin have a strong rhythmic modulation of spike firing in concordance with the large voltage oscillations recorded in AC mode. However, when the DC components are considered, the population of origin is expected to have a weak oscillatory modulation that rides on a large tonic response, as it also has to account for the large (invisible) baseline.

### Large LFPs do not imply a comparable efficiency of the underlying currents on individual units

Large LFPs have attracted much attention simply because they are easy to record, and they have driven researchers to hunt for relationships with many other parameters of brain activity or behavior. Some paradigmatic cases are the hippocampal theta rhythm and the slow cortical oscillations (Timofeev, 2011; Colgin, 2013; Kowalczyk et al., 2013). However, as already mentioned, these LFPs are large due to structural (cytoarchitectonic) factors rather than functional ones. On account of structure, strong synaptic inputs may contribute negligibly to LFPs while others that have a reduced impact on targeted units set up large LFPs. For instance the functionally chief excitatory input from the ipsilateral CA3 to the CA1 produces LFPs 5–10 times smaller than theta oscillations, and the important contralateral input is nearly completely occluded in LFPs (Martín-Vázquez et al., 2016). Meanwhile, powerful basket cell somatic inhibition that governs cell output in the CA1 produces minimal LFPs (Benito et al., 2014). By contrast, distal inhibition of the apical dendrites of these cells gives rise to the large theta rhythm (Ibid; Melzer et al., 2012), whose underlying currents probably serve as modulators for nearby excitatory inputs rather than establishing cell outputs (Herreras et al., 1988). In the case of slow cortical oscillations, the nature of the current generators remains unclear. While the output of most neurons is limited to short intermission epochs (*up states*) when synaptic currents are more abundant and varied, field oscillations are several times smaller (Timofeev, 2011).

### The “Reach” of LFPs does not depend on the amplitude at its origin

**Figure 5:**
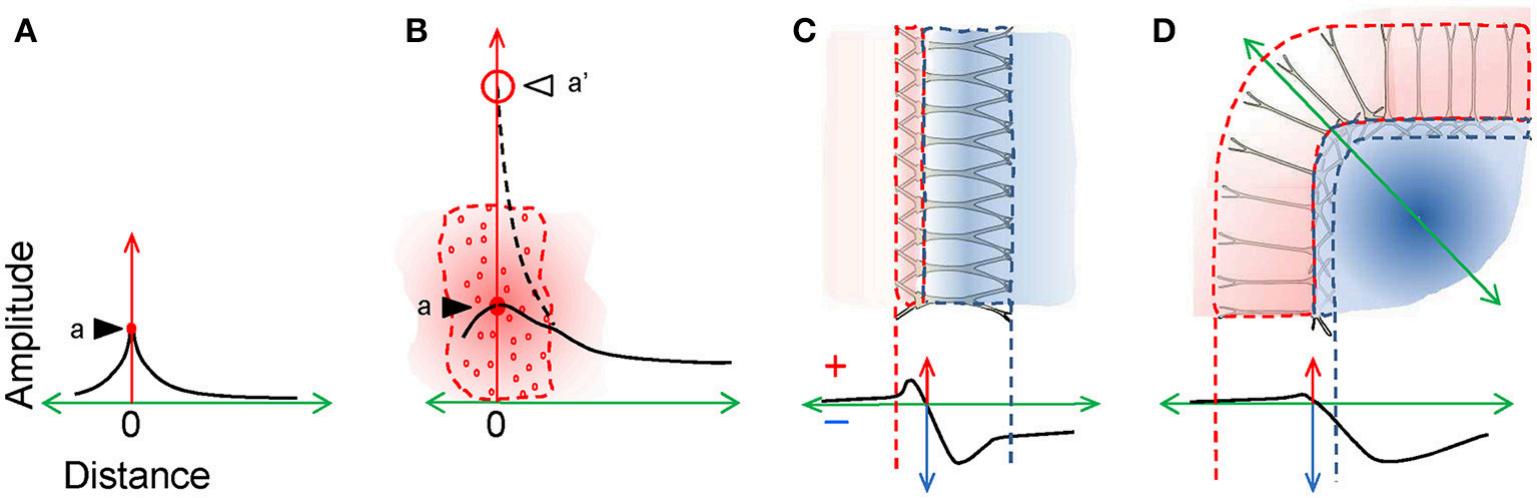
**The configuration of the source at the origin determines the spatial reach. (A)** A point source (dot labelled *a*) produces an electric field that decays exponentially with distance (the black trace represents the associated FP). **(B)** A complex source composed of many elementary point sources (small red circles) behaves differently in the region occupied by them and beyond. The local amplitude of the FP is defined by a site-specific volume average of the total charge density (represented as a single point source *a* in position zero), while the voltage adopts values according to the distribution of actual charges. However, the external portion (volume-conducted currents) behaves as if the charges had been unified in a single point (a' at position zero, and dashed line). Note that local-to-remote decay is far less pronounced than that for a point-source. **(C)** Dipolar currents of neurons produce intense cancellation within the sources and the spatial distribution of the FP is determined by the heterogeneous distribution of local charges. A laminated structure of parallel neurons behaves as a laminar dipole, with maximum positive and negative values typically within the source space (dashed lines in the plot). By contrast, the remote fields vary according to the subcellular location of the inputs and the cell geometry. **(D)** Curved structures produce differential clustering of currents outside the space of the source and the FP may then become larger than inside the area occupied by the source itself.

Simple reasoning might lead one to think that the larger the FPs are at the site of the source, the further away they would spread. However, this is not the case. An essential point to remember is that the distance at which a source is expected to produce measurable fields (the so-called reach or spread) does not depend on the amplitude at the origin but rather, on the spatial features there. It is important not to confound the theoretical decay of an electrical field at a distance from a point source with the decay of FPs from brain sources in a volume (Figures 5A,B). Brain sources are extremely complex and shifty entities from a geometric point of view, and also, they are very heterogeneous in terms of the current density over their own space. Consequently, in order to anticipate the decay of a compound FP over a distance one has to carefully consider its internal spatial configuration, which is defined by the intensive and heterogeneous clustering and cancellation of inward and outward currents. This in turn depends on the morphology and population architecture of activated neurons, as well as on a number of functional factors (for additional discussion: Martín-Vázquez et al., 2016). Intuitively, the net current density in a given volume is only a small fraction of the total current released to the extracellular space, and its composition is hardly predictable. In a figurative manner, one might say that LFPs are extracellular electrical remnants of neuronal activity, i.e., the result of a small amount of net charge that remains unbalanced in a given volume after summing all the inward and outward currents from cell elements contained within. Different sites of the overall source may be populated by different neural elements, and some parts of an activated structure with complex spatial architecture may provide a distinct contribution at a distance than others. Given the heterogeneity of such “left-over” currents, their collective behavior is best estimated using aggregate models of realistic cytoarchitecture (López-Aguado et al., 2002; Lindén et al., 2011; Makarova et al., 2011; Martín-Vázquez et al., 2013, 2016). In our experimental and computational studies we found that the necessary clues can be found in the micro—and the mesoscopic structure of the sources, the most relevant being the spatial extension of the source and its overall charge configuration (dipolar vs. quadrupolar). The former is mostly determined by the spatial coverage of the activated synaptic territory and the latter, by its subcellular distribution and the population architecture (Figures 5C,D). These anatomo-functional considerations may explain why some cortical LFPs are recorded in distant structures despite their local amplitude being moderate or small, and why the giant LFPs recorded in the hilus of the Dentate Gyrus can hardly be appreciated a few hundredths of microns away (Fernández-Ruiz et al., 2013; Martín-Vázquez et al., 2016).

A particular case that has attracted much attention is the discrepant expanse of sensory receptive fields in the cortex measured by LFPs or spikes, which range from a few hundred microns to several millimeters (Gieselmann and Thiele, 2008; Katzner et al., 2009; Denker et al., 2011; Eggermont et al., 2011; Kajikawa and Schroeder, 2011; Gaucher et al., 2012; Liu et al., 2015). Several explanations have been proposed for this phenomenon, such as the size of the brain or the very nature of the LFPs. Thus, the broader fields obtained with LFPs could reflect either volume-conducted or subthreshold synaptic activity, prompting questions regarding the spatial spread of LFPs. Understanding the relationship between LFPs (the inputs) and spikes (the outputs) is obviously of great interest, although the receptive fields are not a proxy for “elementary” current sources nor spike trains can be inferred from LFPs (Rasch et al., 2008). This is particularly the case in complex networks as the cortical column in which LFPs and spikes cannot be traced to the same units. Although cortical receptive fields are small, they are not point sources and hence, they are subject to the uncertainties discussed above. Indeed, the layer-dependent spread of LFPs (Xing et al., 2009) or the distinct feature selectivity reported for LFPs or spikes (Gail et al., 2004; Nielsen et al., 2006; Berens et al., 2008) highlight the differences in the nature and sensitivity of these two parameters. It also indicates that the relationship between them is complex, with a number of interposed biophysical, connective and functional aspects that jointly contribute to the relationships between inputs and outputs on a population scale. For example, recorded LFPs reflect some but not all inputs (Martín-Vázquez et al., 2016) and some, but not others, may be correlated with spike activity (de Cheveigné et al., 2013). In addition, age, experience, training and even ongoing activity modify the receptive fields (Arieli et al., 1996; Fiser et al., 2004), indicating that connectivity varies and hence, the physical structure of the sources too, which may provoke changes in the spatial spread of LFPs or in the relative contributions of network components (Bos et al., 2016). As suggested by many, volume-conduction may indeed play a role in the reported different spatial spread of cortical LFPs and spikes, and provided that all other factors remain the same, one could expect it to be stronger the larger the activated region (Fernández-Ruiz et al., 2013), thereby reaching other cortical and subcortical regions. Therefore, the extent of volume-conduction estimated for LFPs using minimal stimuli cannot be used for complex natural stimuli in which the increased coherence of the inputs translates into larger activated areas, hence a varying proportion of local and volume-conducted FPs at any site. Besides, the obtained estimations apply only for the specific pathway/s and target neurons tested, and cannot be used as a canonical value in all conditions. It can be expected that sources of similar extent but different charge distribution produce different spatial spread of the associated LFPs (see below). The less risky line seems to characterize the features encoded by LFPs and spikes of each cortical region and sensory modality, while the nature of LFPs should be rather examined in function of their own substrate, the sources of current.

### Electrodes have no spatial sensitivity or frequency selectivity

Although the properties of electrodes may have some impact on the signals recorded, the electrodes “sense” (and average) any field potentials that arrive in their vicinity (Nelson and Pouget, 2010). Except for the “ultraslow” (DC) component that requires non-polarizable recording materials, most electrode types and materials pick up slow and fast frequencies similarly within physiologically relevant ranges (0.1 Hz–3 kHz). Rather, it is the local geometry of the source, along with the electric properties of the media that govern the rate of FP decay and therefore, whether it may be recorded at a given distance. Thus, if two sources of similar intensity and location but with different spatial configurations are co-activated, it may well be that only one is sensed by a distant electrode (see below).

### LFPs may be larger at a distance from the source

While it might appear intuitive that an electric field reaches its maximum amplitude close to the source, this does not apply for complex multineuronal sources (Figure 5). The non-regular overall shape of activated structures may produce peak FP values that deviate from the center of mass of the source. In layered structures, the asymmetric morphology of the activated units and/or the subcellular location of the inputs also displace the position of the peak values from the center of the mass (Figure 5C). However, in curved or folded structures, the peak value may fall completely out of the physical space (Figure 5D). This has been shown in the U-shaped hippocampal Dentate Gyrus, where granule cells are all oriented radially with respect to the structure. Such a configuration promotes supernormal clustering of volume-conducted currents on the concave side, which yields LFPs 10–20 times larger than at the source itself (Fernández-Ruiz et al., 2013). Also, the LFPs appear to irradiate asymmetrically toward the open end, a sort of anisotropic effect that should be taken in account when estimating the location and intensity of deep sources from scalp recordings. Similar effects can be expected in other brain structures, such as the cortical gyri, the cerebellum and the curved lateral geniculate nucleus of primates (Makarova et al., 2014).

## Unresolved issues

Most issues dealt with so far concern to the possible errors made by ignoring the critical influence of the geometry of the source or by neglecting to consider technical limitations. A number of unresolved issues regarding the nature of LFPs have been broadly considered in the contemporary literature (mentioned in Technological Developments and the Need for Biophysical Training), but scarcely here. Although the aim here is to highlight the less considered aspects, these can also be introduced into a number of currently ongoing debates where they may offer alternative explanations. As an example, the main concepts on one such issue will be briefly reviewed below.

### Tissue capacitance, monopoles, frequency-filtering, and source geometry

Early researchers considered brain tissue an essentially resistive media, where capacitive, inductive and magnetic effects were deemed negligible (Lorente de Nó, 1947; Mitzdorf, 1985; Nunez and Srinivasan, 2006). This supported the commonly accepted view that FPs in the brain are instantaneous reflections of neural currents, greatly facilitating their mathematical treatment. The issue is now being revisited, although the different approaches employed have offered contradictory results. An exhaustive review is beyond the scope of this article, and thus only some of the relevant findings will be addressed, adding the influence of source geometry as a new element to the debate (for additional discussion see Okada et al., 1994; Bédard et al., 2004; Logothetis et al., 2007; Lindén et al., 2010; Gomes et al., 2016).

The main focus is on elucidating whether the conducting media has substantial capacitive properties that are distinct from those of the neuron membranes. If significant, these properties could modulate the spread of currents in the volume and promote a low-pass filter effect whereby slow waves would reach further than fast ones. Tissue capacitance might also lead to the accumulation of charge of a given sign (monopoles) during neural activity, challenging the instantaneous nature of neural sources and their exclusive dipolar treatment. Such a possibility is supported by the slow movement of ions in the tortuous extracellular space (Syková and Nicholson, 2008) as opposed to the instantaneous nature of electric fields.

The variables that can be gathered in experiments are tissue impedance and voltage gradients, from which capacitance and current density may be derived, respectively. Both these measures are extremely tricky to obtain, and they are associated with numerous pitfalls. Regarding the former, the main causes of error are tissue distortion, overlooked shortcircuiting of the current paths, and inadequate settings and devices (see Li et al., 1968 and López-Aguado et al., 2001 for in-depth discussion). Most studies in brain tissue show little or no differences in tissue impedance for circulating currents within physiological frequencies (0.1–5 KHz) (e.g., Okada et al., 1994; Logothetis et al., 2007; but see also Gomes et al., 2016). Heterogeneous resistivity and its alterations during activity or in association with pathologies are well-known (van Harreveld and Ochs, 1956; Ranck, 1970; López-Aguado et al., 2001). Such phenomena may account for changes in LFP amplitude and may even establish preferred paths for current spread within the tissue, posing a problem for source localization when using distant recordings (e.g., EEG). However, frequency-filtering would not be promoted by heterogeneous tissue resistivity alone. Rather it requires a significant contribution of tissue reactance, and it should be evident as a gradual change of phase in the FPs away from the source (similar to the increasing lag and duration of intracellular potentials by membrane capacitance). Phase changes within the source itself cannot be used for this purpose as these may be originated by a number of different mechanisms, such as the mentioned cable properties, intrinsic currents, or the spatial mixing of fields elicited by two out-phased and displaced sources. Due to experimental difficulties, some authors consider that the lack of direct evidence leaves this question open, even though early literature is full of examples that make this unlikely. For instance, somatosensory evoked potentials elicited by peripheral nerve stimulation produce the so-called standing (far) potentials that drop in amplitude but retain a constant latency and waveform when recorded centimeters away from the source in different parts of the body or the brain (e.g., Cracco and Cracco, 1976; Kimura et al., 1984).

**Figure 6:**
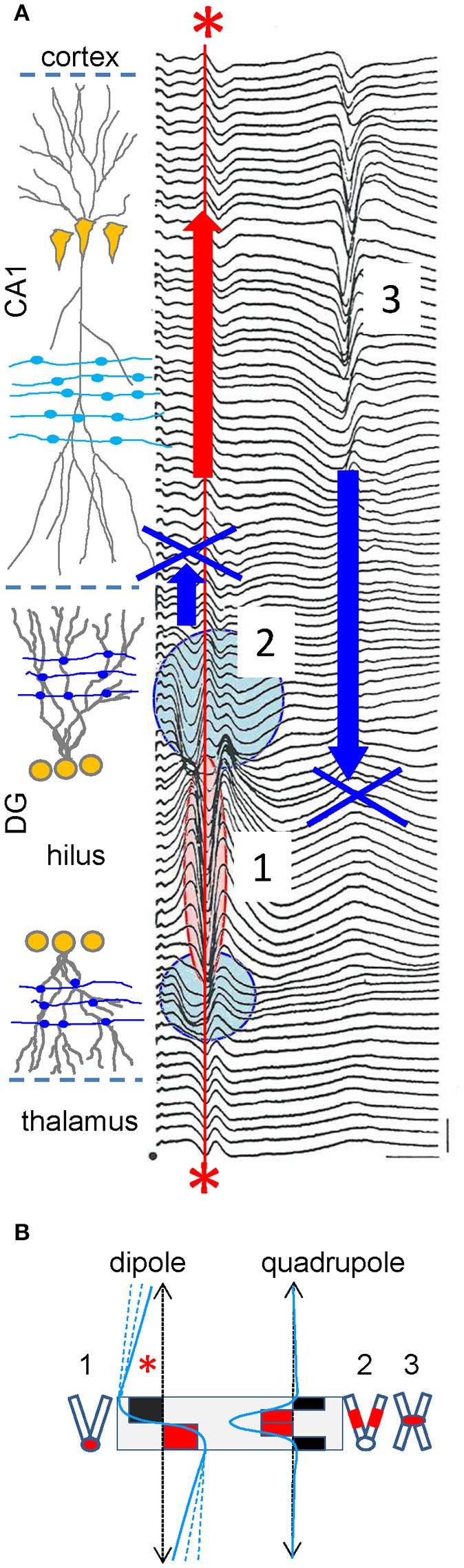
**Experimental example of volume-conduction established by sources with a dipolar or quadrupolar charge distribution. (A)** Spatial profile of evoked potentials recorded with a roving pipette through the hippocampal CA1 and DG regions in response to medial entorhinal cortex activation (step: 20 μM). The fEPSP (2) originated by granule cells is recorded at sites of synaptic contact only, while the monosynaptic population spike (1) is recorded locally and beyond the physical boundaries of the source cells in the hilus (between cell layers) and at distant sites in the CA1, cortex and thalamus (asterisks). By contrast, the trisynaptic pyramidal cell population spike in CA1 (3) is only recorded within the boundaries of the source cells. Modified with permission from Herreras (1986). **(B)** Dipole (left) and quadrupole (right) configurations of the charge distribution (red and black solid squares), and the associated FP profile at a distance (blue lines). The gray box marks the boundaries of the cell generators. The numbered cell dummies depict the cell morphology and zones activated by FPs in **(A)**. Note the reach of dipolar sources (asterisk) compared to the quadrupolar self-contained profiles. The dashed blue lines indicate that FPs decay at lower rates away from the source as they extend further in a plane normal to the graph. In the quadrupolar configuration only the width of the profile is modified inside the source but it hardly changes at a distance from it.

We shall now introduce source geometry into the debate. The argument is often used that extracellular spikes (fast waves) have a shorter reach than the slower FPs, although it is obviously flawed because the sources are different (one or thousands of neurons). In fact, the opposite can be observed when the number of active neurons is equaled (Figure 6A). Thus, the sharp population spike generated by granule cells in the Dentate Gyrus (~0.5–1 kHz) is recorded far from this region as a stationary potential without lag or apparent phase changes. Conversely, the slower field-EPSP (~40–60 Hz) elicited by the same cells hardly extends beyond their physical boundaries (see Fernández-Ruiz et al., 2013 for additional explanations). Hence, the same cells produce different sources of current that reach a different distance in the volume. The explanation for this lies in the configuration of the charge distribution. During spike firing, the population somatic currents in granule cells conform to an extracellular dipole, whilst synaptic currents in mid dendritic locations lead to a quadrupolar (sandwich-like) arrangement, whose far fields are dramatically smaller than the dipolar ones (Figure 6B). Furthermore, unlike granule cells, the somatic spike currents in the nearby pyramidal cells (flanked by basal and apical dendritic trees), establish a quadrupolar configuration. As such, the population spike elicited by this population does not reach far beyond the region of the active membranes. These simple observations lay bare the sheer dominance of source geometry, and more specifically the influence of its internal charge distribution, over other factors that modulate the reach and the features of FPs at a distance from the sources, such as the intensity at origin, the physical boundaries or the frequency.

As it is unlikely that any factor at a distance from a neural source may cause significant frequency-filtering or substantial monopoles the focus turns to its immediate surroundings, in which assuming homogeneous electrochemical properties may not be justified (Ranck, 1963; Gomes et al., 2016). However, direct evidence for this is still missing. Occasional claims for the existence of monopoles of neuronal origin (e.g., Riera et al., 2012) appear to be based on a rigid interpretation of CSD data. Rather, non-zero currents in CSD studies are normally taken as proof of spurious current, particularly since this approach is subjected to numerous experimental uncertainties and assumptions that do not endorse its quantitative use (for additional discussion see Leung, 1979; Mitzdorf, 1985; Herreras, 1990; Gratiy et al., 2013; Martín-Vázquez et al., 2013; Herreras et al., 2015). For instance, CSD is numerically derived from voltage profiles measured at discrete equidistant points, a procedure that weights the current surges with different spatial extent unequally, as is the case for active and return currents along the morphology of neuron generators. Indeed, there is no interpolation technique that can figure out the spatial gradients generated by unknown mixtures of currents with different spatial frequencies.

When considering the local factors that contribute to extracellular FPs, membrane capacitance and intrinsic currents in dendrites are known to modulate the amplitude and duration (i.e., the frequency) of unitary and population membrane currents, or extracellular potentials (Varona et al., 2000; López-Aguado et al., 2002). Some authors suggested that this may explain frequency-filtering (Lindén et al., 2010), yet it is doubtful that such factors may have any significant effect on a mesoscopic scale where the contribution of the different neural elements is averaged in the volume. Some LFPs with a particular dominant frequency can be ascribed to specific strata in laminated structures, although there are no reports of gradual changes to slower frequencies moving away from these.

While the issue is far from settled, we might be advised not to forget the never refuted classic view that multiple sources of reduced extension are subjected to intensive volume (spatial) averaging (Elul, 1971; Nunez and Srinivasan, 2006). Thus, the probability of temporal overlap is smaller for shorter than for longer wavelength events, which converts the degree of correlation of individual currents into a low-pass filter effect at a distance from the sources.

## Concluding remarks: when LFP interpretation is more reliable and when it isn't

Intracerebral local field potentials (LFPs) are easy to record, although interpreting their specific features is not trivial. They are generated by small electric currents that are remnants from population activity, and they have distinct qualitative and quantitative relationships with the participating neurons in different structures. There is no set of rules that can replace the systematic exploration of the sources and a careful consideration of all the technical limitations. Inadequate identification of the sources makes the huge body of cumulated evidence to house controversy. A remarkable number of past and present studies using LFPs as a quantitative index of neural activity are bound to be subjected to future reappraisal as new markers appear. A valid sequence may be (1) discriminating the local or remote origin; (2) determining the simple or composite nature and disentangling the components using spatial (not temporal) discrimination; (3) finding the source neurons producing the current for each component; and (4) identifying the upstream population that establishes the temporal dynamics. These should be completed by finding a baseline, without which a correct quantitative appreciation of temporal fluctuations cannot be safely undertaken. It should be emphasized the relative validity of studies seeking temporal correlations between unit firing and intracellular recordings to LFPs, as they are strongly subjected to causal indetermination. Such studies are of great help but cannot take hold of the ultimate physical nature of the sources, namely, portions of tissue with shifting spatial boundaries and charge distribution. Neurons are at the beginning and the end of LFPs, but the relationships they maintain are complex and varied.

Micro—and mesoscopic structural factors are the main elements governing the essential characteristics of LFPs, such as amplitude, polarity, and extension. In addition, they also configure the temporal pattern on account of the mixing of temporal dynamics from the different co-activated sources at different locations, or even when they arise from the same source neurons. Obtaining the pathway-specific components considerably alleviates the problems described here and helps establish whether they reflect unitary activity, functional assemblies, or mass phenomena. Current techniques already allow their spatial distribution to be defined. The confounders listed here on one hand, and the access to reliable pathway-specific temporal dynamics on the other are both solid arguments indicating that spatial factors are among the most important issues to be explored in depth in the following years.

Understanding that only a few regions, populations and pathways produce LFPs is important for global theories of brain function. LFPs are used to establish preferred relationships between certain networks, or activity in circuits, and they are too often upgraded to major functioning modes of the brain in the literature, while the role of most neuron populations and structures not contributing to LFPs, or more precisely, to rhythmic LFPs, is neglected. Obviously such an oversimplified way of thinking is doomed to failure.

## Author contributions

The author confirms being the sole contributor of this work and approved it for publication.

### Conflict of interest statement

The author declares that the research was conducted in the absence of any commercial or financial relationships that could be construed as a potential conflict of interest.

## Supplementary material

Video 1 **Two-dimensional representation of spatiotemporal variations of field potentials in the volume upon different combinations of synaptic inputs into a realistic aggregate of hippocampal pyramidal cells (see Figure 1)**.
